# Cerebellar Hemangioblastoma Resection Complicated by Postoperative Vasogenic Edema in the Setting of Concurrent Immunotherapy Treatment

**DOI:** 10.7759/cureus.97442

**Published:** 2025-11-21

**Authors:** Aashka Sheth, Nicholas Dietz, Andrea Becerril-Gaitan, Rahim Kasem, Akshitkumar Mistry, Brian J Williams, Dale Ding, Isaac Abecassis

**Affiliations:** 1 Neurosurgery, University of Louisville, Louisville, USA

**Keywords:** cerebellar hemangioblastoma, immunotherapy, oncology, tumor, vasogenic edema

## Abstract

Hemangioblastomas are rare, benign vascular tumors of the central nervous system that are often found in the cerebellum, brainstem, or spinal cord. Common clinical symptoms are related to the mass effect, such as headaches, ataxia, and hydrocephalus. While hemangioblastomas frequently present with vasogenic edema, sustained postoperative edema after tumor resection is an unexpected finding. We report a case of cerebellar hemangioblastoma in a 66-year-old male patient with metastatic prostate cancer and progressive headaches, dizziness, and ataxia. Surgical resection was complicated by significant postoperative edema and worsening of symptoms, requiring urgent reoperation. The patient’s chemotherapeutic regimen included docetaxel and leuprolide, which have both been reported in cases of vasogenic edema. Chemotherapy drugs induce endothelial cell dysfunction and trigger vascular leakage, resulting in vasogenic edema. Additionally, gonadotropin-releasing hormone (GnRH) agonists such as leuprolide may impair cerebrospinal fluid (CSF) regulation, resulting in increased intracranial pressure and subsequent edema. The combination of immunosuppressive drugs and GnRH agonists in this patient’s cancer treatment regimen, along with vascular disruption from the hemangioblastoma itself, may have had a synergistic effect on vasogenic edema development. Our patient’s postoperative edema was successfully managed via decompressive laminectomy and placement of a ventriculoperitoneal shunt, with resolution of symptoms seen postoperatively and at the 12-week follow-up. Tumor surgeons should be advised that patients on chemotherapy and immunotherapy agents may be at higher risk of postoperative edema and an associated mass effect on the surrounding structures.

## Introduction

Hemangioblastomas are rare, generally benign, vascular tumors of the central nervous system (CNS) most likely to be found in the cerebellum, brainstem, or spinal cord [1,2]. These tumors arise sporadically in 75% of cases or as a component of Von Hippel Lindau syndrome, with an average presentation in the fourth to fifth decade of life [1,2]. Clinical presentation varies based on the tumor location. For instance, hemangioblastomas involving the posterior fossa tend to present with progressive neurological symptoms like headaches, ataxia, nausea, and emesis due to the resultant mass effect, cerebrospinal fluid (CSF) flow obstruction, hydrocephalus, and increased intracranial pressure [3]. Surgical excision remains the mainstay treatment with a favorable prognosis in most cases and low recurrence rates [1]. Symptomatic improvement can be seen immediately after surgery, after total resection. If resection is not possible due to the tumor location, radiosurgery may be an appropriate alternative [3,4]. While hemangioblastomas may be associated with preoperative edema and hydrocephalus, most cases are mild, clinically insignificant, and resolve postoperatively [5]. 

In the setting of concomitant use of certain immunotherapy drugs, such as docetaxel, cases of induced vasogenic edema secondary to the drug’s effect of increasing vascular permeability have been reported [6,7]. The gonadotropin-releasing hormone (GnRH) agonist leuprolide has also been reported to have similar effects, as it is a cytotoxic agent [8,9]. Therefore, it is possible that a therapeutic regimen involving both agents may amplify the effect on development of vasogenic edema in the pre- or postoperative settings.

We describe a case of cerebellar hemangioblastoma treated with surgery and gross total resection in a patient with metastatic prostate adenocarcinoma on multiple chemotherapy and immunotherapy agents, complicated by significant post operative cerebellar edema, resulting in brainstem compression and hydrocephalus requiring surgical decompression. 

## Case presentation

**Figure 1:**
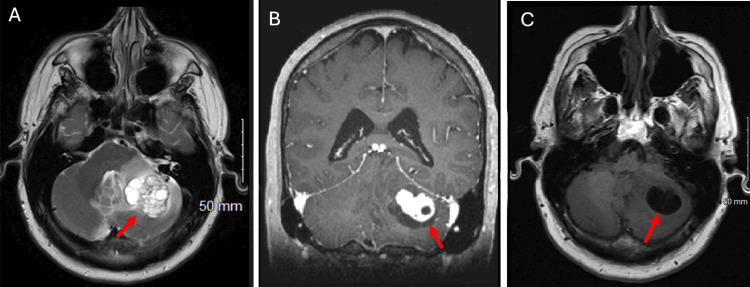
The patient's preoperative magnetic resonance imaging (MRI) T2-weighted axial MRI brain, post contrast showing a large heterogenous mass lesion in left cerebellum (panel A, red arrow) with intensely enhancing peripheral nodules (diameter 33 mm x 34 mm x 27 mm). T1-weighted coronal, post-contrast MRI brain demonstrating extensive vasogenic edema (panel B, red arrow) that extends to the vermis and displacement of cerebellar tonsils. T1-weighted axial, post-contrast MRI brain showing hypointense cystic lesion (panel C, red arrow) causing mass effect on the fourth ventricle and vermis.

A 66-year-old male patient with a history of stage IV prostate adenocarcinoma presented to the emergency department with two months of progressive headaches, dizziness, and feeling off-balance. The patient had been diagnosed with prostate adenocarcinoma with extra-prostatic extension, bilateral pelvic and inferior abdominal lymphadenopathy, and widespread osseous metastases eight months prior. The patient had a Gleason score of 4+5, indicating that his prostate adenocarcinoma was highly aggressive and poorly differentiated based on histopathological examination. He was started on triple therapy with leuprolide acetate, darolutamide, and docetaxel four months prior to this hospital admission. Outpatient brain magnetic resonance imaging (MRI) demonstrated a new heterogeneously enhancing left cerebellar mass with extensive vasogenic edema (Figure 1).

On initial physical exam, the patient's speech was fluent, and all the cranial nerves were intact. Motor strength was 5/5 in bilateral upper and lower extremities with some left-sided dysmetria, and sensation was intact throughout. Hoffman’s sign was negative. Karnofsky Performance Status was 100, indicating a full functional activity status with no impairments in daily living.

**Figure 2:**
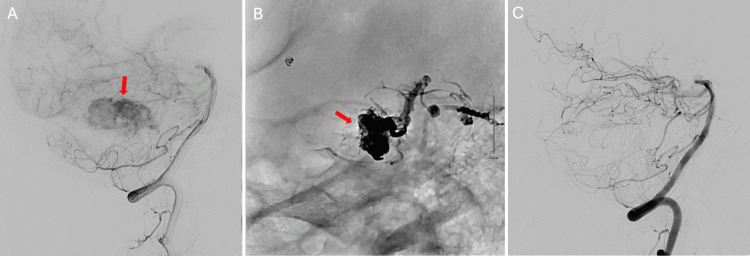
The patient's preoperative diagnostic angiography Lateral view of left vertebral artery demonstrating tumor predominantly supplied by distal branches of the left superior cerebellar artery (panel A). Magnified lateral view of left SCA branches post-embolization (panel B). Lateral view of left vertebral artery showing decrease in tumor blush after successful embolization with Onyx 18, Coils, and n-butyl cyanoacrylate (n-BCA; panel C).

A diagnostic angiography was performed to assess the hypervascularity associated with the lesion and the possibility of preoperative embolization (Figure 2).

**Figure 3:**
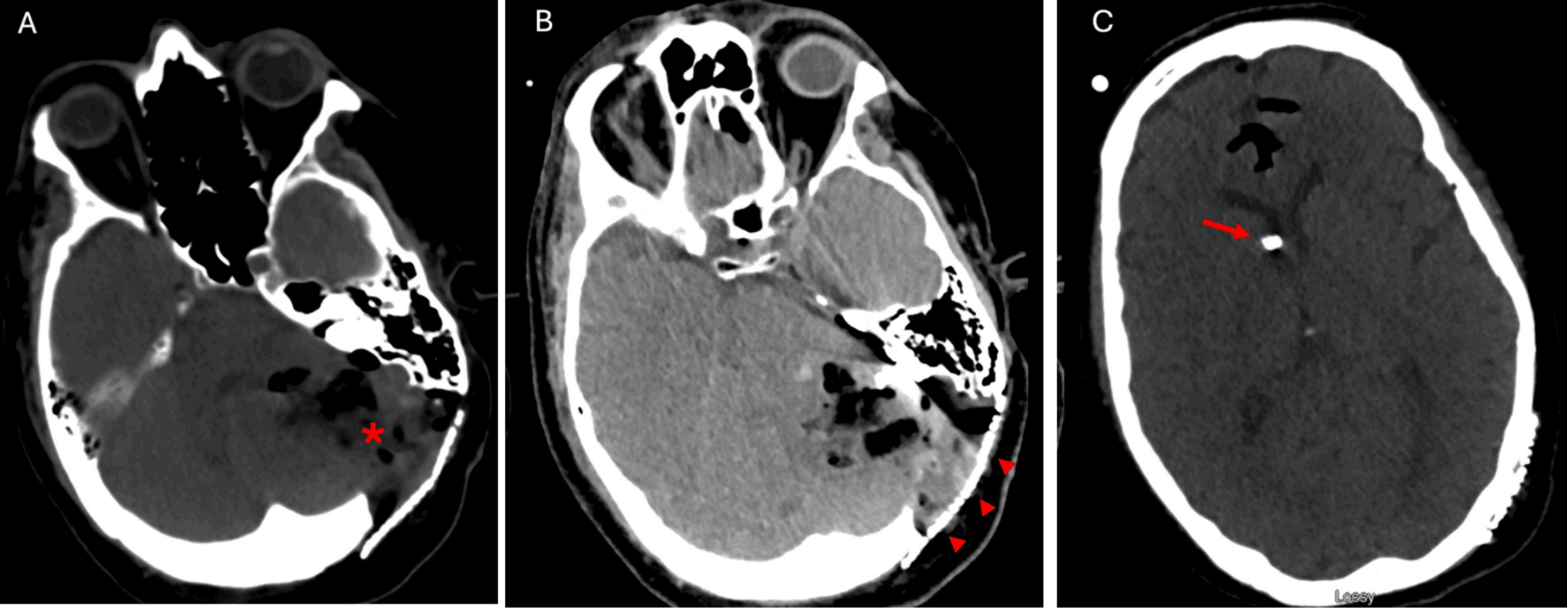
The patient's postoperative computed tomography of the head (CTH) Non-contrast axial CTH demonstrating interval removal of left cerebellar mass lesion with surgical bed predominantly occupied by multiple gas pockets (panel A, red asterisk) post suboccipital craniectomy with mesh cranioplasty (panel B, red arrowhead). Placement of right frontal external ventricular drain, with the tip terminating in the lateral ventricle near the right foramen of Monro (panel C, red arrow).

Multiple branches of the left superior cerebellar artery were seen to be supplying the tumor and were subsequently embolized. The patient was subsequently taken to the operating room for an external ventricular drain (EVD) placement and left retrosigmoid craniotomy (Figure 3).

**Figure 4:**
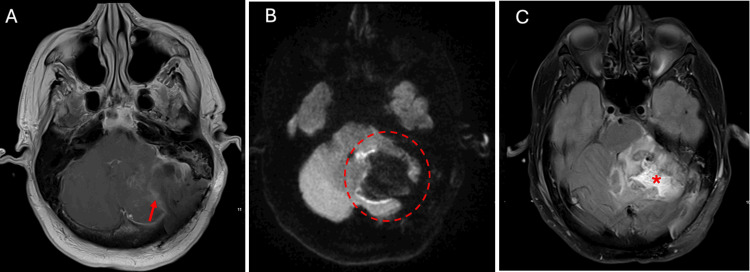
The patient's postoperative brain magnetic resonance imaging (MRI) T1-weighted post contrast axial MRI demonstrating gross total resection (panel A, red arrow) with minimal residual blood products. Diffusion-weighted imaging (DWI) showing resection cavity with no evidence of acute postoperative stroke (panel B, red circle). T2 Fluid-attenuated inversion recovery (FLAIR) axial MRI showing post-op edema (panel C, red asterisk) causing significant mass effect on the brainstem, which is stable compared to the preoperative scan.

Intraoperatively, stealth navigation was used for EVD placement. The patient was then placed in three-point Mayfield pins and transverse and sigmoid sinuses were identified and a paramedian linear incision was made over the left transverse sinus. Multiple burr holes were made to expose the transverse sinus and inferior margin of the sigmoid sinus. A retrosigmoid craniotomv was fashioned and the dura and pia were incised. The cerebellum was decompressed by draining the peritumoral cyst. The tumor was exposed, and the vessels surrounding the tumor were coagulated circumferentially. A large piece of tumor was dissected and resected, and care was taken to remove any residual tumor (Figure 4).

Cautery, irrigation, and hemostasis were used to achieve this. After ensuring no residual tumor remained, DuraGen (Integra LifeSciences, Princeton, NJ, USA) and titanium mesh (Stryker, Michigan, USA) were placed. The wound was irrigated with vancomycin and subsequently closed. Pathologic examination of the tumor confirmed a diagnosis of hemangioblastoma, WHO grade I [10]. 

**Figure 5:**
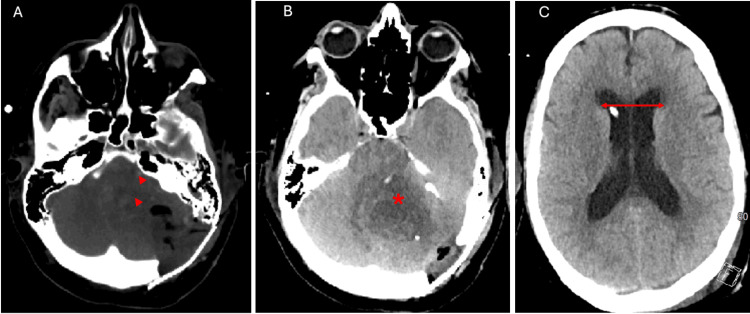
The patient's computed tomography of head (CTH) on postoperative day two Non-contrast axial CTH demonstrating pneumocephalus and some parenchymal petechial hemorrhages (panel A, red arrowheads). Increased postoperative vasogenic edema (panel B, red asterisk) causing significant effacement of fourth ventricle and posterior fossa cisterns. Increased size in lateral ventricles with frontal approach external ventricular drain (EVD) at the right frontal horn (panel C).

On the postoperative day one, the patient developed worsening headache, dysarthria, and left cranial nerve VI palsy. A computed tomography (CT) head scan (Figure 5) demonstrated cerebral edema with progressive effacement of the fourth ventricle, with worsening of an acquired Chiari malformation-type presentation.

**Figure 6:**
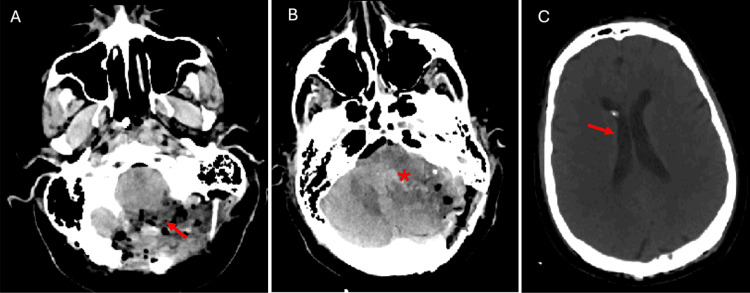
The patient's computed tomography of head (CTH) post suboccipital craniectomy and C1 laminectomy Non-contrast axial CTH demonstrating postsurgical sequela from suboccipital craniectomy and removal of old titanium mesh with C1 laminectomy. A small volume of subcutaneous gas overlying the operative site with extension to the posterior neck is demonstrated (panel A, red arrow). A similar degree of vasogenic edema is appreciated with continued effacement of fourth ventricle and posterior fossa cisterns (panel C, red asterisk). There is a significant decrease in size of the lateral ventricles with frontal approach external ventricular drain (EVD; panel C, red arrow).

The patient was initially monitored on high dose steroids but clinical symptoms worsened, including a pneumonia presumably due to aspiration and lower cranial nerve dysfunction, as well as CT scans showing progressive effacement of the fourth ventricle. An urgent suboccipital craniectomy with removal of the titanium mesh and Chiari decompression via C1 laminectomy were performed on postoperative day three (Figure 6).

The patient subsequently had objective improvements in speech and diplopia with cranial nerve VI deficits resolved. Upon attempting EVD weaning, the patient had worsened headaches and dysarthria, so a ventriculoperitoneal (VP) shunt was placed. The patient was discharged to an acute rehabilitation facility on postoperative day 20. At the six-week follow up, the patient displayed continued clinical improvement from his condition at the time of hospital discharge, endorsing some mild dysarthria and ataxia. He will continue to follow up with speech and physical therapy, with reassessment of imaging and clinical condition in three months.

## Discussion

We report a case of cerebellar hemangioblastoma resection in a 66-year-old male patient with metastatic prostate cancer on multiple chemotherapy and immunotherapy agents, who developed significant postoperative vasogenic edema and Chiari-like symptom presentation, requiring subsequent suboccipital decompression.

Hemangioblastomas are commonly associated with preoperative vasogenic edema secondary to increased vascular permeability of tumor vessels and high interstitial pressure within the tumor, resulting in interstitial fluid buildup in areas surrounding the tumor [11,12]. As this process is driven by intrinsic factors of the tumor, this is seen as a reversible process that often subsides with tumor removal. One study found that 84% of patients with hemangioblastomas had associated edema with postoperative hydrocephalus, resolving without further intervention in 94% of cases [5]. Larger hemangioblastomas may have associated brainstem edema or hemorrhage postoperatively, with a possible mechanism being loss of autoregulation resulting in normal perfusion breakthrough, but this risk is reduced by preoperative embolization [13]. However, given that the patient underwent preoperative embolization and still had significant cerebral edema and fourth ventricle effacement postoperatively suggests a particularly unusual amount of postoperative edema. 

A plausible explanation for edema development in this patient could be due to the immunotherapy for metastatic prostate cancer, specifically docetaxel. Chemotherapeutic or immunosuppressive drugs can induce endothelial cell dysfunction and trigger vascular leakage, resulting in vasogenic edema [6]. Certain chemotherapeutic agents can disrupt the integrity of the blood-brain barrier, resulting in complications such as edema, hemorrhage, or ischemia [7]. Multiple immunotherapy drugs, including docetaxel, have been implicated in the development of posterior reversible encephalopathy syndrome (PRES), a condition with subcortical vasogenic edema resulting in headaches, encephalopathy, and visual disturbances [6,14]. Although PRES does not commonly present in the cerebellum or brainstem, our patient’s vasogenic edema potentially developed due to a similar mechanism as docetaxel-induced PRES. A report of docetaxel-induced hydrocephalus proposed that docetaxel’s effect on microtubules resulted in ependymal cilia disruption and impairment of CSF clearance [15].

Additionally, our patient was on leuprolide, a GrRH agonist frequently used to treat prostate cancer [9]. Although the mechanism is unclear and likely multifactorial, there have been reports of increased posterior and subcortical vasogenic brain edema development in patients taking GnRH agonists [8,9]. GnRH agonists can have cytotoxic effects on vascular endothelial cells, resulting in endothelium damage and loss of tight junctions [8]. Estrogen has been found to have protective effects on endothelial cells, and GnRH agonists decrease estrogen levels, disrupting this effect [9]. There have also been multiple reports of idiopathic intracranial hypertension in patients after the use of leuprolide, suggesting that GnRH agonists may impair CSF regulation, resulting in increased intracranial pressure and subsequent edema [16-18]. Therefore, the combination of immunosuppressive drugs and GnRH agonists in this patient’s cancer treatment regimen, along with vascular disruption from the hemangioblastoma itself, may have had a synergistic effect on vasogenic edema development. 

Of note, steroids are commonly used to treat vasogenic edema through decreasing inflammation and increasing tight junction expression [19]. It has been reported that steroids take 48-72 hours for symptoms to abate [20]. Our patient was given high-dose steroids after the first operation, but his vasogenic edema persisted and worsened within 24 hours, requiring urgent intervention. Further studies should elucidate optimum timing of steroid administration for maximum benefit in cases of expanding vasogenic edema. Surgical decompression represents a successful management strategy for cases of vasogenic edema unresponsiveness to steroids. 

## Conclusions

We present a case of cerebellar hemangioblastoma in a patient with metastatic prostate cancer in which surgical resection was complicated by significant postoperative edema. While associated edema is common in patients with hemangioblastoma, sustained edema after tumor removal is an abnormal finding. Our patient’s use of chemotherapeutic and hormone modulating drugs likely resulted in increased vascular permeability and symptomatic vasogenic edema, which was successfully managed through surgical decompression. Tumor surgeons should be advised that patients on chemotherapy and immunotherapy agents may be at higher risk of postoperative edema and associated mass effect on the surrounding structures. Multidisciplinary preoperative evaluation or closer postoperative monitoring may be required in this patient population to minimize postoperative complications. 
